# *Lactobacillus paracasei* L9 Improves Amino Acid Absorption in Aged Mice via Enhanced LAT2 Expression Through the Akt/mTOR Pathway

**DOI:** 10.3390/nu18091468

**Published:** 2026-05-04

**Authors:** Wenhao Li, Lili Qiu, Qianqian Huang, Ran Wang, Rui Song, Yixuan Li, Xiaoyu Wang

**Affiliations:** 1 Key Laboratory of Precision Nutrition and Food Quality, Department of Nutrition and Health, China Agricultural University, Beijing 100193, China; liwenhao0730@163.com (W.L.); wangran@cau.edu.cn (R.W.); 2 College of Food Science & Nutritional Engineering, China Agricultural University, Beijing 100083, China17508934475@163.com (Q.H.); songrui_123@cau.edu.cn (R.S.)

**Keywords:** probiotic, aging, amino acid absorption, gut microbiota, butyric acid

## Abstract

**Background:** Aging is frequently accompanied by disrupted amino acid homeostasis, contributing to malnutrition and physical function decline in elderly individuals. However, age-related impairment of intestinal absorptive capacity limits the efficacy of protein supplementation alone to improve nutritional status. Gut microflora diversity is regarded as a key factor for nutrient absorption. *Lactobacillus paracasei* L9 (L9) has recently been shown to improve gut health, but its effects on intestinal amino acid absorption remain unclear. **Methods:** In this study, 15-month-old male C57BL/6J mice (*n* = 4 per group) were administered L9 (4 × 10^10^ CFU in 100 μL) via daily gavage for 9 months to investigate the effect of L9 on amino acid absorption. **Results:** After L9 intervention, both plasma amino acid levels and the expression of large neutral amino acid transporter 2 (LAT2), a key transporter associated with neutral amino acid absorption, were significantly increased. Subsequently, microbiota composition analysis revealed that L9 modulated the gut microbiota, increasing the abundance of multiple beneficial bacteria in aged mice. Targeted metabolomics then revealed that plasma concentrations of butyric acid significantly elevated in aged mice after L9 intervention. Furthermore, IEC-6 cells experiments were conducted, demonstrating that butyric acid can enhance LAT2 expression via activating the Akt/mTOR pathway. **Conclusions:** Overall, these results suggested that L9 significantly enhanced amino acid absorption capacity in aged mice via the butyric acid-Akt/mTOR-LAT2 axis, and this identifies potential targets for improving age-related malnutrition and providing a promising strategy to maintain amino acid homeostasis in the elderly.

## 1. Introduction

Proteins are essential for human health, facilitating tissue repair and serving as precursors for enzyme and hormone synthesis. It is noteworthy that protein deficiency has become a significant public health issue within the elderly population. Several studies have noted that approximately 5% to 10% of elderly individuals suffer from protein-energy malnutrition [1]. Such nutritional deficiencies increase the incidence of sarcopenia, impaired immune function, and simultaneously heighten the risk of morbidity and mortality in the elderly [2,3]. Thus, enhancing protein utilization and amino acid absorption capacity are crucial for the health of the elderly.

The current strategies to improve protein utilization in the elderly are limited. Protein or amino acid supplementation is commonly used to improve protein deficiency [4]. However, decreased amino acid transporters in the elderly could limit the efficiency of protein or amino acid supplements. Also, relevant studies have reported that excessive protein or amino acid supplementation may lead to multiple issues, including decreased expression of intestinal amino acid transporters, reduced appetite, and shortened lifespan [5]. Therefore, endogenous regulation of the digestive and absorptive capacity could be an effective strategy to improve the protein utilization of the elderly.

Impaired digestive and absorptive capacity was considered as a major factor contributing to protein deficiency in the elderly [6]. Research has demonstrated that age-related gastric dysfunction is characterized by reduced gastric acid secretion, lowering efficiency of protein digestion [7]. In addition, pancreatic tissue volume decreases significantly with aging [8,9]. Such pancreatic atrophy impairs secretory function, thereby limiting the production and release of digestive enzymes. Previous studies have reported that pancreatic secretion in the elderly drops to one-third of the average level in adults [10]. Other research has also found reduced secretion of proteases such as trypsin and chymotrypsin among older adults, with trypsin activity declining to two-thirds of the normal reference value [11,12]. This leads to oligopeptides and free amino acids delivered to the small intestine decreasing. Moreover, amino acid absorption efficiency is impaired due to decreased expression of amino acid transporters. Many studies have shown that the expression levels of amino acid transporters, including B0-type amino acid transporter 1 (B^0^AT1), large neutral amino acid transporter 1 (LAT1) and Taurine Transporter, decline with age [7,13,14]. Importantly, our previous research suggested that large neutral amino acid transporter 2 (LAT2) might play a role in the decreased transportation of neutral amino acids in the elderly [15]. Thus, LAT2 is a key target for enhancing enhance amino acids absorption in the elderly.

With advancing age, the gut microbiota undergoes significant restructuring in both humans and animal models. In the elderly, aging is characterized by reduced microbial diversity and an increased proportion of pro-inflammatory bacteria, a condition known as gut dysbiosis, which is closely linked to age-related decline [16]. Similarly, aging reduces the alpha diversity of gut microbiota in mice, accompanied by decreased abundance of short chain fatty acid (SCFA)-producing bacteria and enrichment of pathogenic bacteria [17]. Gut microbiota diversity is widely recognized as a critical determinant of nutrient absorption [18]. The absorptive function of the intestinal tract is improved by intestinal microflora through the metabolism of dietary components, modulation of immune responses, and preservation of intestinal barrier integrity. Multiple previous studies demonstrated that probiotics enhance the absorption efficiency of various nutrients, including amino acids, fatty acids and minerals, by improving intestinal barrier integrity and transporter function [19,20,21]. *Lactobacillus paracasei* L9 (L9), a commensal bacterial strain originally isolated from the feces of healthy centenarians [22,23], has been demonstrated to exert multiple protective effects on intestinal health, including alleviating post-weaning diarrhea in piglets [24], enhancing butyric acid-producing capacity to suppress the IL-6/STAT3 pathway and thus ameliorate the progression of colitis [25], and strengthening intestinal immune function by upregulating serum Immunoglobulin G and mucosal Immunoglobulin A levels in mice [26]. However, the potential of L9 to improve nutrient absorption remained to be elucidated.

In this study, a nine-month intervention with L9 in aged mice was conducted. Then, the impact of L9 intervention on amino acid absorption capacity in aged mice, and its underlying mechanism, were investigated, offering a novel strategy for improving age-related amino acid imbalance.

## 2. Methods

### 2.1. Animal Study

**Figure 1:**
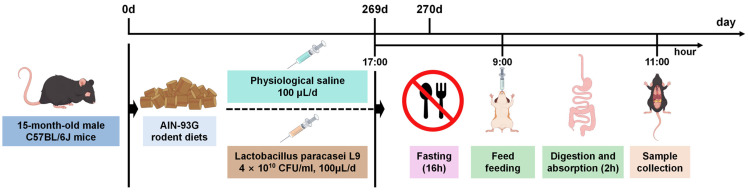
Animal experiment flow chart.

Male C57BL/6J mice aged 15 months old (Vital River, Beijing, China) were housed in a specific pathogen-free environment under a 12 h light/dark cycle. They were randomly assigned to two groups: natural aging (24 m group, *n* = 4) receiving 100 μL physiological saline (Solarbio, Beijing, China), and L9 intervention (L9 group, *n* = 4) receiving an equal volume of 4 × 10^10^ CFU/mL L9 suspension (Fucheng Biotechnology, Sanhe, Hebei, China) via daily gastric lavage in the afternoon (Figure 1). All procedures were approved from the Ethics Committee of China Agricultural University (AW32504202-5-8), and mice were euthanized after nine months.

### 2.2. Apparent Total Tract Digestibility

Apparent total tract digestibility (ATTD) was assessed by the Kjeldahl method as previously described [27]. Briefly, fecal samples were oven-dried to constant weight and then ground into a fine powder. Then, 300 mg of powder were divided into equal portions for acid digestion. The digestion solution comprises 6 g of potassium sulphate, 0.5 g of copper sulphate, and 10 mL of concentrated sulfuric acid. The resulting digestate was distilled with sodium hydroxide to convert nitrogen into ammonia, which was captured in boric acid solution and subsequently titrated with standard hydrochloric acid for total nitrogen quantification. Crude protein was calculated using a conversion factor of 6.25.

### 2.3. Determination of Plasma Amino Acids

Plasma amino acid concentrations were determined as previously described [28]. Briefly, plasma amino acids were analyzed using a nanoAcquity UHPLC system (Waters, Milford, MA, USA) interfaced with a TripleTOF 7600 mass spectrometer (AB SCIEX, Framingham, MA, USA). Separations were achieved on a BEN Admine column (Waters, USA) at 45 °C. Mobile phase A comprised water with 20 mmol/L ammonium acetate and 0.5% formic acid; a mixed solvent of acetonitrile and water in an 85/15 volume ratio served as mobile phase B. Subsequently, the gradient program commenced with a flow rate of 0.3 mL/min and a 5 μL injection volume. Multiple reaction monitoring data were collected and processed using SCIEX OS software (Version 3.4.5, AB SCIEX, Framingham, MA, USA).

### 2.4. Western Blotting

Protein extraction was performed according to a published protocol [15]. Briefly, samples were lysed in RIPA buffer containing protease (PMSF) and phosphatase inhibitor cocktails. Protein concentrations were determined using the BCA Protein Assay Kit (Thermo Fisher Scientific, Waltham, MA, USA). Protein was separated by gel electrophoresis and transferred onto polyvinylidene difluoride (PVDF) membranes via electrotransfer. After blocking, anti-β-actin antibody (4967S, Cell Signaling Technology, Danvers, MA, USA), anti-LAT2 antibody (UM500058, Origene, Rockville, MD, USA), anti-Akt antibody (bsm-52010R, BIOSS, Beijing, China), anti-p-Akt antibody (bsm-52030R, BIOSS, China), anti-mTOR antibody (HA500126, HUABIO, Hangzhou, Zhejiang, China), and anti-p-mTOR antibody (HA600094, HUABIO, China) were incubated overnight at 4 °C. After incubating with the appropriate HRP-conjugated secondary antibodies, image analysis software (SAI600, GE Healthcare Life Sciences, Little Chalfont, England, UK) was employed to detect the luminescent signals of proteins. For protein quantity analysis in the whole cells, β-Actin was used as the housekeeping protein. Density measurements were calculated using Image J (Version 1.54, National Institutes of Health, Bethesda, MD, USA).

### 2.5. Immunohistochemistry

The immunohistochemical experiment was performed as previously described [29]. After hydration of the sections, antigen retrieval was performed in Tris-EDTA buffer (pH 9.0), and endogenous peroxidase was quenched with hydrogen peroxide. Then, sections were incubated overnight at 4 °C with anti-LAT2 antibody (UM500058, Origene, China). Subsequently, sections were incubated with horseradish peroxidase-conjugated secondary antibody, followed by DAB staining (Zhongshan Jinqiao Biotechnology Co., Ltd., Zhongshan, Guangdong, China). Sections were observed under an optical microscope, and multiple regions were randomly selected from each slide. The positive area was quantified by Image J.

### 2.6. 16S rRNA Gene Sequencing

The measurement of 16S rRNA gene sequencing followed the previously established protocol with minor modifications [30]. Sequencing was performed on an Illumina NovaSeq 6000 platform (2 × 250 bp paired-end) by Majorbio Bio-Pharm Technology Co., Ltd. (Shanghai, China). In brief, fresh fecal samples were collected in sterile, enzyme-free tubes, and bacterial DNA was isolated using the PowerSoil DNA Isolation Kit (Qiagen, Hilden, NRW, Germany). The V4 region of 16S rRNA was PCR-amplified, and libraries were constructed for Illumina sequencing according to standard protocols. High-quality libraries were run on the NovaSeq 6000 platform. Raw data underwent quality filtering to remove low-quality reads (Q < 20, length < 50 bp), chimeras, and mismatched paired-end overlaps (<5 bp). Amplicon sequence variants (ASVs) were generated via UNOISE denoising, and their representative sequences were taxonomically classified using QIIME2 (version 2023.5). All subsequent bioinformatic analyses were performed using the Majorbio Cloud Platform (https://cloud.majorbio.com, accessed on 6 May 2025).

### 2.7. Targeted Metabolomics Analysis

The detection of short-chain fatty acids followed the previously established protocol [31]. In brief, fecal samples (100 mg) were spiked with 2-ethylbutyric acid internal standard, extracted with methanol per Majorbio protocol, and analyzed by Agilent 8890B-5977B GC–MS (AB SCIEX, Framingham, MA, USA) on an HP-FFAP column; peaks were auto-identified with MassHunter and quantified against a linear curve. Mouse plasma metabolomics used an ExionLC–QTRAP 6500+ UHPLC-MS/MS (Agilent Technologies, Santa Clara, CA, USA) with a Waters BEH Amide HILIC column (35 °C, 6 min, 1 mL/min acetonitrile/formate gradient, ESI+ 5500 V); Sciex OS auto-integrated peaks and concentrations were read from standard curves.

### 2.8. IEC-6 Culture

IEC-6 cells were cultured as previously described [32]. Briefly, Dulbecco’s Modified Eagle’s Medium supplemented with 10% foetal bovine serum and 1% penicillin/streptomycin was employed for IEC-6 cells’ cultivation. Subsequently, cells were collected after incubation for 24 h in media containing 0–100 mM sodium butyrate for subsequent experiments.

### 2.9. Statistical Analysis

Statistical analysis was performed in SPSS 26.0 (IBM, Armonk, NY, USA), with statistical significance defined as *p* < 0.05. Asterisks denote significance levels: * *p* < 0.05, ** *p* < 0.01, and *** *p* < 0.001. Following verification of variance homogeneity, multiple group comparisons were analyzed by one-way ANOVA with LSD post hoc testing. Graphs were generated in GraphPad Prism 8.4.3 (GraphPad Software, San Diego, CA, USA), and data are expressed as means ± SEM.

## 3. Results

### 3.1. L9 Intervention Enhances Protein Utilization in Aged Mice

**Figure 2:**
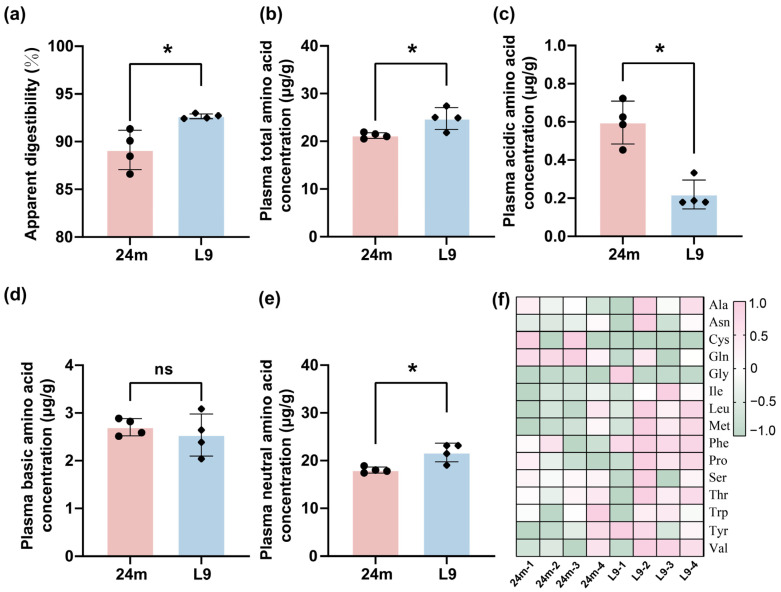
Circulating amino acid profile was reshaped in aged mice with L9 supplementation. (**a**) Apparent total intestinal digestibility in mice; (**b**) plasma concentration levels of total amino acid concentration; (**c**) plasma concentration levels of acidic amino acid concentration; (**d**) plasma concentration levels of basic amino acid concentration; (**e**) plasma concentration levels of neutral amino acid concentration; (**f**) heatmap of all neutral amino acids. Data were analyzed by one-way ANOVA for *n* = 4 individual experiments. Significance differences are indicated by *, corresponding to *p* < 0.05, ns, no significant difference.

Amino acid homeostasis imbalance is common in the elderly, which brings a clinical challenge for the treatment of osteoporosis and sarcopenia. To investigate whether L9 could ameliorate age-related defects in amino acid utilization, a 9-month intervention study was conducted in aged mice. ATTD analysis revealed a significant increase in the L9 group compared with the 24 m group (Figure 2a), indicating improved protein digestion and greater amino acid release in the intestine of amino acids in the intestinal tract. Based on this finding, the circulating amino acid pool was analyzed, and a significant increase in the total plasma amino acid concentration was observed in the L9 group (Figure 2b). To elucidate the specific effects of L9 on different categories of amino acids, amino acids were grouped according to their standard biochemical classification into neutral, acidic, and basic types for subsequent analysis. The results demonstrated that the level of plasma neutral amino acids was significantly elevated in aged mice subjected to L9 intervention (Figure 2e,f). In contrast, the content of plasma acidic amino acids was significantly decreased in aged mice treated with L9, while no significant change was observed in that of plasma basic amino acids compared to 24 m group (Figure 2c,d). Collectively, these findings indicated that long-term L9 supplementation could enhance the overall amino acid absorption in aged mice, with a more pronounced effect on neutral amino acids, and the underlying mechanisms of this effect required further investigation.

### 3.2. LAT2 Expression Levels in the Intestines of Mice Following L9 Intervention

**Figure 3:**
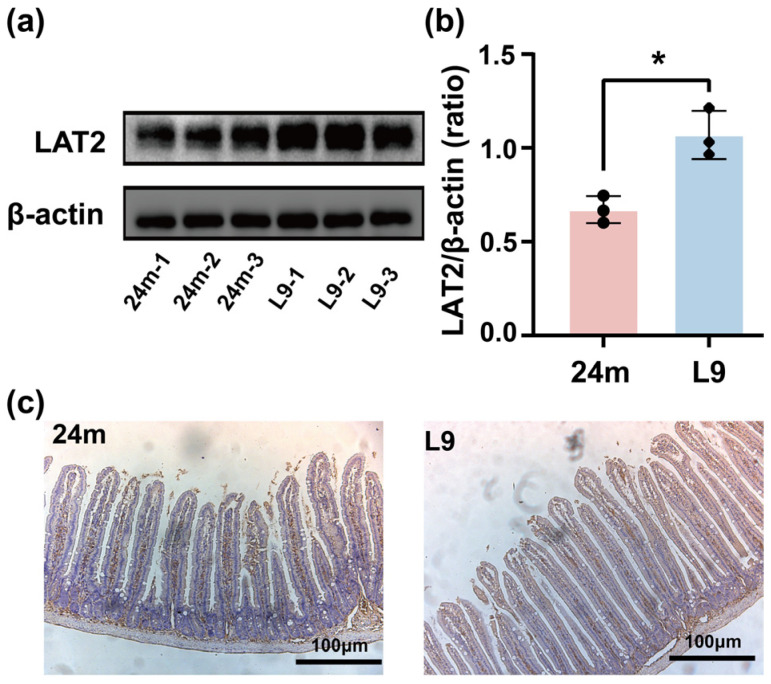
LAT2 expression in the duodenum of aged mice was upregulated after L9 intervention. (**a**) Representative Western blot images of duodenal LAT2 protein levels; (**b**) quantitative analysis of LAT2 protein expression normalized to β-actin. Data were analyzed by one-way ANOVA for *n* = 4 individual experiments. Significance levels were indicated by *, corresponding to *p* < 0.05. (**c**) Representative immunohistochemical staining of LAT2 in duodenum (the brown area in the figure).

Intestinal amino acid transporters (AATs) play a crucial role in amino acid absorption. LAT2 is a key determinant of amino acid absorption efficiency in the elderly. Therefore, the expression levels of LAT2 in duodenal mucosal scrapings from aged mice were analyzed by Western blotting. Western blotting analysis revealed that the protein expression of LAT2 in the treatment group was significantly higher than that in the age-matched control group (Figure 3a,b), indicating the recovery of LAT2 expression level. This increase was further confirmed by immunohistochemical analysis, which showed that the staining intensity detected for LAT2 in the duodenum was significantly enhanced in the L9 group compared with the aged control group, with no alteration in the cellular localization of the protein (Figure 3c). These results indicate that the marked increase in neutral amino acid absorption was mediated by the upregulated expression of LAT2 in the L9 group.

### 3.3. Analysis of the Gut Microbiome Composition in Mice Following L9 Intervention

**Figure 4:**
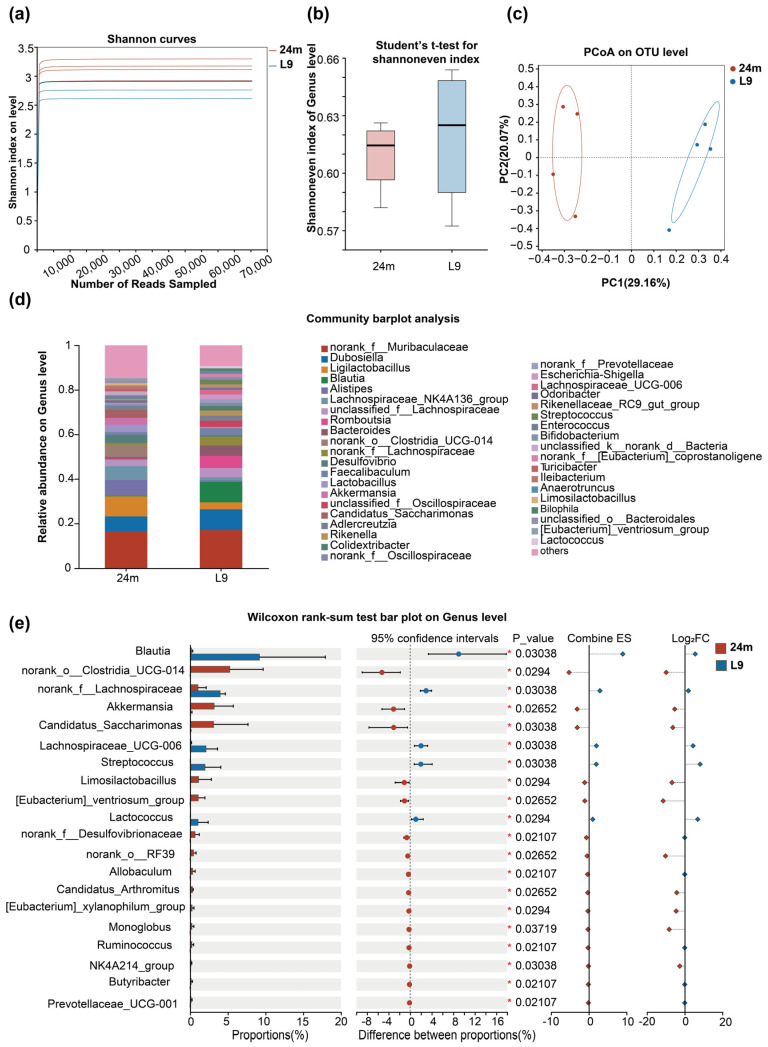
L9 reshaped the gut microflora of aged mice. (**a**) ASU dilution curve; (**b**) α diversity index; (**c**) principal coordinate analysis; (**d**) relative abundance of genera in fecal communities analyzed; (**e**) analysis of significantly differentially abundant taxa. Data were analyzed by one-way ANOVA for *n* = 4 individual experiments. Significance levels are indicated by *, corresponding to *p* < 0.05.

To elucidate the potential mechanism by which L9 promotes amino acid absorption in aged mice, the alterations of the gut in aged mice with L9 supplement were investigated. Fecal microbial composition was examined via 16S ribosomal RNA (rRNA) GENE sequencing, and rarefaction curve analysis confirmed sufficient sequencing depth for all samples (Figure 4a). Alpha diversity was analyzed to evaluate the effect of L9 supplementation on the gut richness in aged mice. No significant effect of L9 supplementation on species diversity was observed in the gut of aged mice (Figure 4b). In contrast, β-diversity analysis based on principal coordinate analysis (PCoA) revealed a distinct separation between the L9 and control groups (Figure 4c), indicating that L9 intervention significantly altered the composition of the gut microbiota in aged mice. The results demonstrated that following L9 intervention, there was a significant increase in beneficial bacteria such as *Blautia*, *Faecalibaculum* and *Anaerotruncus* in aged mice (Figure 4d,e). However, the cumulative abundance of several potential pathogens, including *Desulfovibrio*, *Enterococcus*, and *Escherichia-Shigella*, exhibited an opposite trend (Figure 4d,e). In conclusion, our findings collectively demonstrate that the gut microbiota composition of aged mice underwent significant restructuring following L9 intervention.

### 3.4. Short-Chain Fatty Acid Profiles in Feces and Plasma

**Figure 5:**
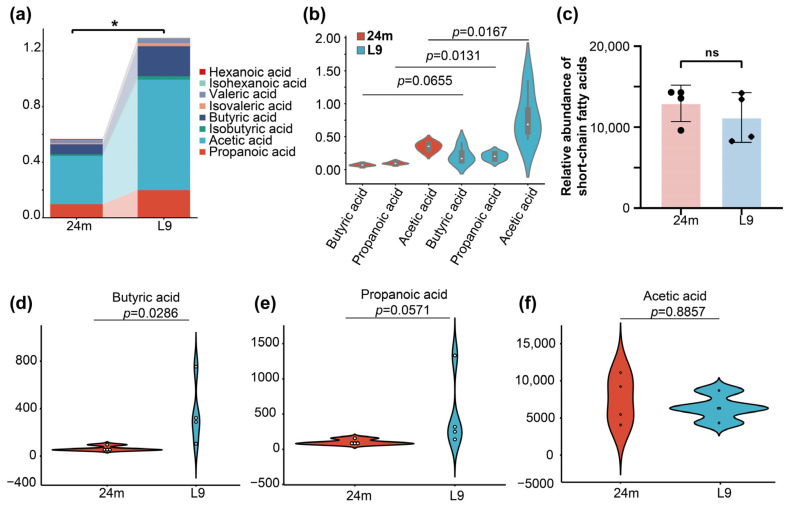
L9 elevates fecal and plasma short-chain fatty acids in aged mice. (**a**) Total SCFA content in feces from aged mice; (**b**) relative abundances of butyric acid, propionate and acetate in feces; (**c**) relative abundance of short-chain fatty acids in plasma; data were analyzed by one-way ANOVA for *n* = 4 individual experiments. Significance levels were indicated by *, corresponding to *p* < 0.05. (**d**) Plasma butyric acid concentration; (**e**) plasma propanoic acid concentration; (**f**) plasma concentration levels of acetic acid concentration. Data were analyzed by two-tailed Mann–Whitney U test for *n* = 4 individual experiments. Significance levels were indicated by *, corresponding to *p* < 0.05.

Short-chain fatty acids (SCFAs) constitute the primary metabolites of gut probiotics, which play a pivotal role in conferring a multitude of health benefits to the host, ranging from maintaining intestinal barrier integrity to regulating immune responses and energy metabolism. Therefore, we investigated the effects of L9 on SCFA metabolism, and targeted metabolomics analysis was performed on fecal and plasma samples from aged mice. As illustrated in Figure 5a, total SCFA content was significantly elevated in the feces of aged mice pf L9 group compared with the control group, with butyric acid, propionic acid, and acetic acid exhibiting significant increases (Figure 5b). This suggested an enhancement of fermentative capacity of the gut microflora in the L9 group. Further analysis of plasma SCFAs levels revealed that L9 administration specifically and significantly raised circulating concentrations of butyric acid in aged mice (Figure 5c–f). These findings demonstrated that intestinal SCFAs production and elevated plasma butyric acid levels were increased by L9, which may contribute to its positive effects on amino acid absorption in aged mice.

### 3.5. Regulation of LAT2 Expression by Sodium Butyrate in IEC-6 Cells

**Figure 6:**
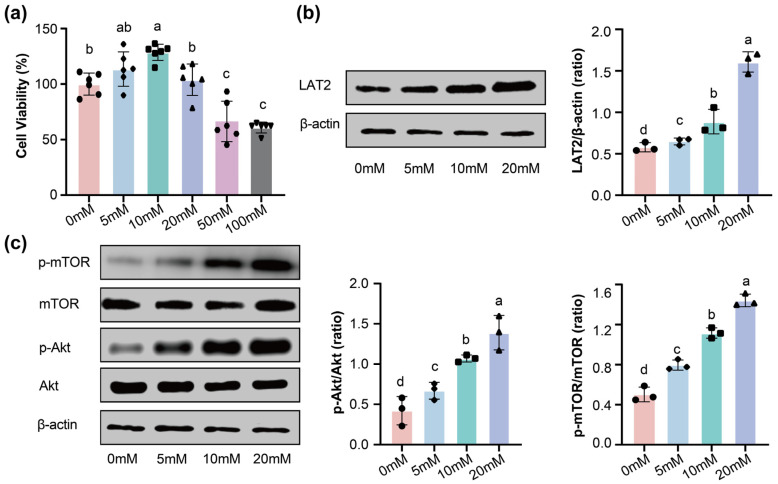
Sodium butyrate upregulated LAT2 in IEC-6 cells via Akt/mTOR signaling. (**a**) CCk8 cytotoxicity test of IEC-6 with 0, 5, 10, 20, 50, 100 mM sodium butyrate, data were analyzed by one-way ANOVA for *n* = 6 individual experiments; (**b**) representative Western blotting bands showed the protein level of LAT2; (**c**) representative Western blotting bands showed the protein level of Akt, p-Akt, mTOR and p-mTOR; Data were analyzed by one-way ANOVA. Different lowercase letters (a, b, c, d) indicate significant differences among groups (*p* < 0.05). CCk8: *n* = 6 per group; Western blot: *n* = 3 per group.

To investigate the regulatory effects of butyric acid on amino acid absorption capacity, the rat intestinal epithelial cell line IEC-6 was utilized as an in vitro model, and the regulatory mechanism of butyric acid on LAT2 expression was subsequently examined. First, the effect of sodium butyrate on IEC-6 cell viability was assessed by CCk8 assay. Cell viability was significantly reduced at sodium butyrate concentrations of 50 and 100 mM (Figure 6a). Based on this finding, a concentration was selected for subsequent experiments to further investigate whether SCFAs regulate LAT2 expression (0–20 mM). It was found that LAT2 expression increased in a dose-dependent manner in response to sodium butyrate stimulation (Figure 6b). Given that the Akt/mTOR pathway is recognized as a central regulator connecting nutrient sensing to transporter expression and is known to be responsive to SCFAs signaling, its activation status was subsequently investigated. Thus, the expression levels of key proteins in the Akt/mTOR pathway including Akt, p-Akt, mTOR and p-mTOR were analyzed. Results showed that the Akt/mTOR pathway was activated by sodium butyrate, with elevated expression levels of p-Akt and p-mTOR (Figure 6c). These results indicated that sodium butyrate could enhance LAT2 expression by activating the Akt/mTOR pathway.

## 4. Discussion

Impaired protein utilization leading to disrupted amino acid homeostasis is common in the elderly. However, amino acid supplementation fails to improve protein utilization efficiency in the elderly. Thus, alleviating age-related decline in intestinal absorption is expected to be an effective intervention strategy. Probiotics are regarded as a potential effective intervention, by which intestinal absorption function could be improved through the modulation of gut microflora diversity. The present study demonstrates that L9 exerts intestinal protective effects by modulating gut microbiota diversity, with the potential to promote amino acid absorption. Multiple previous studies have indicated that probiotics could enhance the absorption efficiency of various nutrients, including amino acids, fatty acids, and minerals, through the improvement of intestinal absorption function [19,20,21]. These results are consistent with the findings of this study, which demonstrated that intestinal amino acid absorption capacity was significantly improved in aged mice following L9 intervention, with particularly pronounced effects observed on neutral amino acid absorption.

Amino acid transporters are crucial for intestinal amino acid absorption. Extensive research has established that aging is associated with downregulation of key amino acid transporter expression [7,14]. Recently, Song et al. [15] reported that reduced LAT2 was a determinant of impaired protein utilization during aging. Restoring intestinal absorption is key to improving amino acid homeostasis imbalance in the elderly. Probiotics could regulate the gut microflora, which plays a crucial role in maintaining health and the digestion, absorption, and metabolism of nutrients. Multiple studies have reported that probiotics could enhance the expression of various intestinal amino acid transporters, including LAT1 B^0^AT1, and PepT1 [33,34,35]. These reports support our findings that L9 intervention enhances the expression of LAT2 in the intestines of aged mice, thereby increasing amino acid absorption.

Probiotics could improve gut health by regulating the abundance of beneficial bacteria within the gut, thereby indirectly improving amino acid absorption. Our research reveals that L9 modulated the gut of aged mice, through elevating the relative abundance of *Blautia*, *Faecalibaculum* and *Anaerotruncus*, which is consistent with numerous studies [36,37]. Furthermore, probiotics maintain gut health by fermenting indigestible carbohydrates within the intestine to produce short-chain fatty acids (SCFAs). SCFAs serve as the preferred energy source for colonic epithelial cells. An adequate energy supply helps maintain intestinal barrier function, preventing pathogens and harmful substances from entering the plasma stream and reducing systemic inflammation risk [38]. Our findings revealed that plasmatic SCFAs including butyric acid and propanoic acid were increased in aged mice with L9. Butyric acid plays a vital role in intestinal health and nutrient absorption. Previous studies have demonstrated that butyric acid supplementation significantly improved protein digestibility and elevated plasma total amino acid levels in pigs and chickens [39,40]. Consistently, the expression and transport activity of peptide transporter 1 (PepT1) in mouse intestine were found to be markedly enhanced by butyric acid, which was accompanied by increased amino acid absorption [41]. This is consistent with the findings that L9 intervention enhanced amino acid absorption capacity in aged mice by upregulating LAT2 expression through elevated plasma butyric acid levels.

Butyric acid is involved in modulating critical physiological processes such as energy metabolism, intestinal barrier integrity, and cell proliferation and differentiation, thereby achieving precise regulation of target genes encoding nutrient transporters, tight junction proteins and other functional proteins [42]. These effects are mediated by its regulation on multiple core signaling pathways, including Akt/mTOR, Wnt/β-catenin and NF-κB. Notably, the Akt/mTOR signaling pathway serves as a critical hub for nutrient sensing and growth regulation within cells, playing a central role in controlling protein synthesis [43]. This regulatory effect occurs through the activation of mTOR, the core effector of the Akt/mTOR signaling pathway, which induces the downstream phosphorylation of ribosomal protein S6 kinase 1 (S6K1) and eukaryotic translation initiation factor 4E-binding protein 1 (4E-BP1), two key substrates mediating the translation regulation of AATs related genes [44]. Studies indicate that butyric acid activated the Akt/mTOR pathway by stimulating the membrane receptors GPR41/43 [45,46], which aligns with our experimental findings. In addition, the activated Akt/mTOR pathway could induce the expression of LAT1, a homologous protein of LAT2, which corroborates our findings [47,48]. These results suggest that L9 alters gut microbiota composition in aged mice, leading to increased plasma butyric acid levels that may activate the Akt/mTOR pathway to enhance LAT2 expression and potentially improve amino acid absorption.

## 5. Conclusions

This study suggests that *Lactobacillus paracasei* L9 (L9) can modulate amino acid absorption capacity in aged mice. Intervention with L9 significantly elevated duodenal LAT2 expression levels, intestinal beneficial bacterial abundance, and plasma butyric acid levels in aged mice, which was associated with enhanced amino acid absorption. This improvement appeared to involve L9 regulation of the butyric acid-Akt/mTOR-LAT2 axis. Overall, this study provides insights into the potential mechanism underlying elevated amino acid absorption associated with L9, offering novel approaches for preclinical intervention.

## Data Availability

The raw data supporting the conclusions of this article will be made available by the authors upon request.
